# A mathematical model for incorporating biofeedback into human postural control

**DOI:** 10.1186/1743-0003-10-14

**Published:** 2013-02-02

**Authors:** Tulga Ersal, Kathleen H Sienko

**Affiliations:** 1 Department of Mechanical Engineering, University of Michigan, 2350 Hayward St, Ann Arbor, MI 48109, USA; 2 Department of Biomedical Engineering, University of Michigan, 2200 Bonisteel Blvd., Ann Arbor, MI 48109, USA

**Keywords:** Biofeedback, Sensory augmentation, Sensory substitution, Mathematical modeling, Postural control, Vibrotactile feedback, Multidirectional perturbations, Vestibular, Balance

## Abstract

**Background:**

Biofeedback of body motion can serve as a balance aid and rehabilitation tool. To date, mathematical models considering the integration of biofeedback into postural control have represented this integration as a sensory addition and limited their application to a single degree-of-freedom representation of the body. This study has two objectives: 1) to develop a scalable method for incorporating biofeedback into postural control that is independent of the model’s degrees of freedom, how it handles sensory integration, and the modeling of its postural controller; and 2) to validate this new model using multidirectional perturbation experimental results.

**Methods:**

Biofeedback was modeled as an additional torque to the postural controller torque. For validation, this biofeedback modeling approach was applied to a vibrotactile biofeedback device and incorporated into a two-link multibody model with full-state-feedback control that represents the dynamics of bipedal stance. Average response trajectories of body sway and center of pressure (COP) to multidirectional surface perturbations of subjects with vestibular deficits were used for model parameterization and validation in multiple perturbation directions and for multiple display resolutions. The quality of fit was quantified using average error and cross-correlation values.

**Results:**

The mean of the average errors across all tactor configurations and perturbations was 0.24° for body sway and 0.39 cm for COP. The mean of the cross-correlation value was 0.97 for both body sway and COP.

**Conclusions:**

The biofeedback model developed in this study is capable of capturing experimental response trajectory shapes with low average errors and high cross-correlation values in both the anterior-posterior and medial-lateral directions for all perturbation directions and spatial resolution display configurations considered. The results validate that biofeedback can be modeled as an additional torque to the postural controller without a need for sensory reweighting. This novel approach is scalable and applicable to a wide range of movement conditions within the fields of balance and balance rehabilitation. The model confirms experimental results that increased display resolution does not necessarily lead to reduced body sway. To our knowledge, this is the first theoretical confirmation that a spatial display resolution of 180° can be as effective as a spatial resolution of 22.5°.

## Background

Biofeedback can be used to supplement or replace missing sensory information by providing the user with information via a functioning sensory modality. To date, individuals with vestibular deficits and older adults have demonstrated improved balance when using electrotactile [1-6], vibrotactile [7-19], auditory [20-25], or multimodal [26,27] feedback displays of body motion during quiet or perturbed standing and gait tasks. For example, vibrotactile feedback of torso tilt has experimentally shown reductions in the root-mean-square sway in subjects with unilateral and bilateral vestibular loss during both linear and rotational single-axis [10,11] and multidirectional [14,28] perturbations of stance. Research is now underway to evaluate biofeedback, also referred to as sensory substitution or sensory augmentation, as a real-time balance aid and as a tool for balance rehabilitation.

Biofeedback-related research aimed at improving balance has primarily used experimental rather than mathematical methods to study the relationship between non-native feedback channels and postural control. As demonstrated by numerous physiological studies [29-38], mathematical models can complement experimental work by allowing for design evaluation and optimization prior to human subject testing, explaining experimental findings and identifying dominant underlying physiological mechanisms.

The literature discusses different mathematical models of human posture with varying levels of complexity. The simplest model used to describe bipedal postural control is an inverted pendulum with feedback control [29-31], which is a one-link representation capturing a single degree of freedom. For example, Peterka used this model to explain the experimental differences in the sensorimotor control systems observed in healthy subjects and subjects with vestibular deficits [29]. When a more sophisticated representation is needed, the number of links can be increased to capture additional degrees of freedom. For example, Barin adopted multiple regression techniques and concluded that a two-link model is sufficient to explain changes in center of pressure (COP) during postural control [32]. Kuo combined a two-link model with a full-state-feedback human sensorimotor control model and optimal control theory to study responses to small perturbations in the anterior-posterior (AP) direction [33]. Kooij et al. developed a three-link model based on optimal estimation theory to characterize the contribution of multisensory information to standing balance and concluded that a predictive element in the controller is essential to compensate for neural time delays [34]. Unfortunately, all of these models only capture movement in the AP direction. Thus, Winter considered two separate models for movement in the AP and medial-lateral (ML) directions [35]; yet, even his models do not capture the dynamic coupling between the AP and ML motions, which is often significant during multidirectional perturbations. Finally, none of the models mentioned above incorporate biofeedback.

To understand how biofeedback affects postural control, Goodworth et al. developed a vibrotactile biofeedback model for a single-link inverted pendulum model of stance [16]. In the single-link model, the ankle angle (which is controlled only through ankle torque) represents the combined kinematics of a number of body segments, i.e., the relative angle between the feet and legs as sensed by the proprioceptive system, the orientation of the head in space as sensed by the vestibular system, the relative position of the head with respect to the environment (which can be important in eyes-open scenarios although not considered in eyes-closed studies such as [16]), and the orientation of the torso in space as measured by the biofeedback device. Because single-degree-of-freedom models have only one sway angle to measure and only one joint torque to control, representations of the sensory integration and control processes associated with biofeedback are relatively straightforward. Although effective at capturing unidirectional postural responses to small perturbations, single-link models represent movement only along a single axis and are limited in their ability to capture multidirectional postural responses (especially during large perturbations). Furthermore, Goodworth et al.’s representation of biofeedback is not readily scalable to models with higher degrees of freedom.

This paper describes a new method to incorporate biofeedback into a multi-degree-of-freedom model for human balance. Rather than considering an integration on the sensory side, the method integrates biofeedback after the existing postural control mechanism generates joint torques and before they are actually applied to the joints. This scalable method is independent of the model’s degrees of freedom, how it handles sensory integration, and the modeling of its postural controller. The model is validated against existing experimental data [28] to demonstrate its ability to replicate the experimentally observed average response trajectories of individuals with vestibular deficits for three different perturbation directions and three different feedback device display configurations. The model is then used to predict the performance of a fourth display with a lower spatial resolution than those experimentally evaluated. Although this study features a vibrotactile feedback display, the unique approach presented herein has the flexibility to describe auditory, visual, electrotactile, and multimodal feedback displays and scale to single or multi-segmented models of human stance, thereby rendering it suitable for a wide range of balance and balance rehabilitation applications.

## Methods

**Figure 1:**
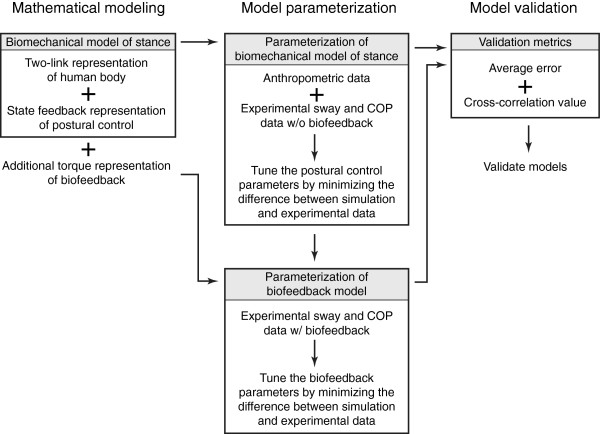
Procedural schematic of the methodology employed in this study.

The procedure followed in this study is outlined in Figure 1 and explained in detail in this section.

### Mathematical modeling

#### Biofeedback

Typical biofeedback systems consist of a motion or force sensor to detect body kinematics or kinetics, respectively, a processor to estimate body kinematics or COP, and a feedback display to provide the user with an additional channel of information. This study assumed that the biofeedback signal was either given in cardinal directions (i.e., in the AP and ML directions) by the device, or was decomposed by the individual into these cardinal directions. Regarding the latter, if the biofeedback signal was given at an angle of *θ* measured clockwise from the navel as viewed in the transverse plane, we assumed that the individual decomposed the signal into its sagittal and coronal components according to cos *θ* and sin *θ*, respectively.

Accounting for experimental observations that individuals control balance in the sagittal and coronal planes independently [18,39], the biofeedback signal was also assumed to be processed and utilized independently in the cardinal directions. Specifically, additional torque signals **T**_*F*_^*S*^ and **T**_*F*_^*C *^were assumed to be generated due to biofeedback independently for sagittal and coronal planes, respectively, as follows:

(1) $$T_{F}^{S}\left(s\right)=\frac{K_{F}^{S}}{\tau_{F}s+1},\;T_{F}^{C}\left(s\right)=\frac{K_{F}^{C}}{\tau_{F}s+1}$$

where **T**_*F*_^*S*^ and **T**_*F*_^*C *^are the additional sagittal and coronal biofeedback torques, respectively, and are vectors with a dimension equal to the number of joints in the model. Thus, they are scalars in a single-link inverted pendulum representation of the body that models only the ankle joint, whereas they become three-dimensional vectors in a model that considers ankle, knee, and hip joints. The variable *τ*_*F *_is the time constant associated with the reaction to the feedback. It was assumed to be the same in both sagittal and coronal directions; hence the superscript *S* or *C* to differentiate the sagittal and coronal plane is omitted. The variable *s* is the Laplace variable, a standard notation for representing transfer functions in the frequency domain. **K**_*F*_^*S *^and **K**_*F*_^*C *^are the steady state magnitude vectors of sagittal and coronal torques due to feedback; i.e., **T**_*F*_^*S*^ = **K**_*F*_^*S *^and **T**_*F*_^*C*^ = **K**_*F*_^*C *^in the steady state.

**Figure 2:**
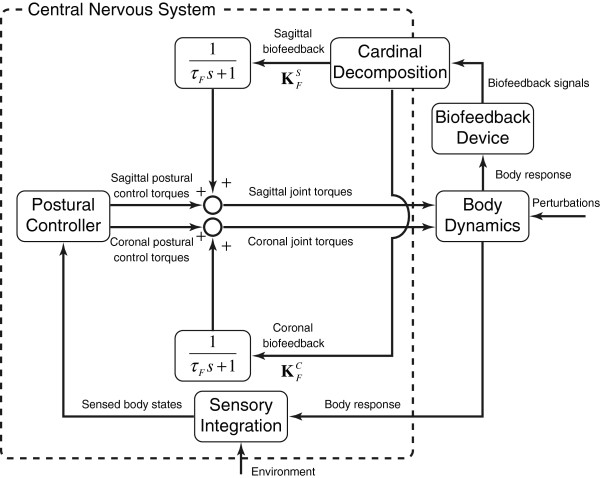
**Block diagram representation of the biofeedback model.** The variable *s* is the Laplace variable.

The biofeedback torques **T**_*F*_^*S *^and **T**_*F*_^*C *^were added to the joint torques generated by a postural controller as follows

(2) $$T_{J}^{S}=T_{\mathrm{PC}}^{S}+T_{F}^{S},\;T_{J}^{C}=T_{\mathrm{PC}}^{C}+T_{F}^{C}$$

where **T**_*J *_represents the vector of joint torques and **T**_*PC *_represents the torque vector generated by the postural controller model. Figure 2 illustrates the integration of this biofeedback model into a generic feedback-based human postural control model. Note that the integration of biofeedback in this approach is independent of the manner in which the human body, sensory integration, and postural control are modeled, and thus is easily applicable to multi-degree-of-freedom models without any constraints on the mathematical representations of the sensory and control systems.

For validation purposes, the proposed modeling framework was applied to experimental results for a representative biofeedback device as follows. The device used an inertial measurement unit to detect body motion and a vibrotactile display comprising 48 vibrating actuators (subsequently referred to as tactors) to provide a feedback signal related to the measured torso tilt. The direction *θ* of the feedback signal was measured clockwise from the navel and was equal to the torso tilt direction (azimuth), which was computed based on the arctangent of the AP and ML tilt components. The device feedback algorithm was such that the magnitude of the feedback signal was equal to the tilt angle (inclination) plus half the tilt angle rate-of-change (see [14] for details). Direction was displayed using as many as 16 columns of tactors based on a “nearest neighbor” principle in which the column closest to the actual tilt direction is activated. The columns were placed around the torso at 22.5° intervals starting from the navel. Within each column, three rows of tactors encoded three different levels of feedback magnitude; no tactors were activated within a 1° dead zone and tactor activation progressed from the bottom to the top tactor row in a stepwise manner as sway increased. Tilt magnitude was modeled based on the algorithm used during the experimental studies (see [14] for details). **K**_*F*_^*S *^and **K**_*F*_^*C *^were expressed as a function of the activated row *r* and the feedback direction *θ*

(3) $$K_{F}^{S}\left(r,\theta \right)=\{\begin{array}{ll} k_{1}^{S}\cos\theta & r=1\\ k_{2}^{S}\cos\theta & r=2\\ k_{3}^{S}\cos\theta & r=3 \end{array},\;K_{F}^{C}\left(r,\theta \right)=\{\begin{array}{ll} k_{1}^{C}\sin\theta & r=1\\ k_{2}^{C}\sin\theta & r=2\\ k_{3}^{C}\sin\theta & r=3 \end{array}$$

where **k**_***i***_, *i* = {1, 2, 3} for the three rows, are constant vectors. To ensure that biofeedback torque monotonically increased with the activated row, the elements of **k**_*i*_ are constrained as *k*_1,*j*_ ≤ *k*_2,*j*_ ≤ *k*_3,*j*_. Here, *k*_*i,j *_refers to the *j*^th^ element of the vector **k**_*i*_.

Sienko et al. showed that this vibrotactile feedback device quickens subjects’ return to upright and reduces sway following a discrete surface perturbation [28]. Furthermore, they varied the number of active tactor columns to determine the effect of spatial resolution on postural performance [14,18,28]. For example, in a 3×4 (3-row, 4-column) display configuration, only four of the tactor columns (navel, spine, and left and right sides) were active, providing 90° spatial resolution. No significant differences were observed among the display configurations, i.e., four columns were as effective as sixteen columns [28].

#### Bipedal stance

The human body was modeled as a two-link inverted pendulum in three-dimensional space, where one link represented the legs and the other link represented the upper body. The feet were not considered; rather, the body was assumed to be connected to the perturbation platform (described below) through the ankles.

Standard vector second-order differential equations were used to express the rigid body dynamics of the two links [40]. Translational dynamics for each link were given by the following concise vector second-order equation

(4) $$F=m\dot{v}$$

where **F** is the total external force acting on the link and **v** is the velocity of the center of mass of the link with respect to the inertial frame. Rotational dynamics were given by

(5) $$T=I\dot{\omega }+\omega \times \mathrm{I\omega}$$

where **T** is the total external torque acting on the link, **ω** is the absolute angular velocity, and **I** is the moment of inertia matrix for the link coordinate frame at the center of mass.

The ankles and hips were modeled as ideal spherical joints. No passive stiffness or damping effects were considered in the joints, as the role of passive torque during stance has been reported to be negligible [41]. The knees were locked due to the fact that the perturbations were small. This also helped reduce the degrees of freedom in the model.

The postural control mechanism was assumed to comprise two independent linear state-feedback controllers for the AP and ML directions. The assumption of independence is supported by experimental studies [18,39], and the linear state-feedback control assumption is standard in the literature [33,34]. State feedback was assumed to be without noise, but with delay. No feedforward or estimation mechanisms were considered. The equations for the postural controllers had the same form in both the AP and ML directions, given by

(6) $$T_{\mathrm{PC}}\left(t\right)=-K\;x\left(t-t_{d}\right)$$

where $T_{\mathrm{PC}}\left(t\right)={\left[\begin{array}{ll} T_{\mathrm{PCa}}\left(t\right) & T_{\mathrm{PCh}}\left(t\right) \end{array}\right]}^{T}$ is the vector of postural control torques at the ankle and hip, **K** is a 2×4 matrix of gains, and $x\left(t-t_{d}\right)={\left[\begin{array}{llll} \alpha_{a}\left(t-t_{d}\right) & \alpha_{h}\left(t-t_{d}\right) & {\dot{\alpha }}_{a}\left(t-t_{d}\right) & {\dot{\alpha }}_{h}\left(t-t_{d}\right) \end{array}\right]}^{T}$ is the state vector of ankle and hip angles and angular velocities with a time delay of *t*_*d*_.

Sensory integration was neglected for simplicity. The states from the human body model were directly fed into the postural controller; i.e., the Sensory Integration block in Figure 2 was not used.

The model was implemented in 20-Sim (Controllab Products B.V., Enschede, the Netherlands). Inputs to the model were the discrete surface perturbations used in [28], which are described next.

### Discrete surface perturbations

**Figure 3:**
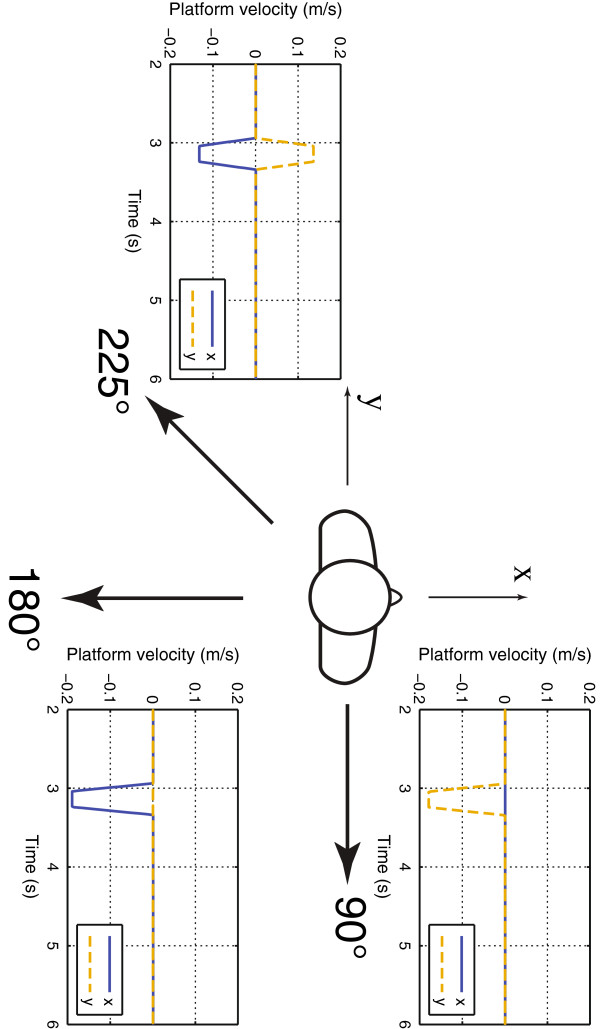
**Perturbations.** Bird’s-eye view showing the perturbation directions, the positive directions for the *x*- and *y*-axes, and the platform velocity inputs for each perturbation direction.

Perturbations were applied using a custom-built 2.1 m^2^ platform [42,43] that could move in an earth-horizontal plane by independent control of motion in two orthogonal (*x* and *y*) directions. Figure 3 shows the platform velocity inputs for the three perturbation directions considered in this study (90°, 180°, and 225°). Perturbations comprised a constant acceleration phase for 100 ms, followed by 200 ms constant velocity and 100 ms constant deceleration phases. The perturbation magnitude was subject specific and ranged from 50 to 70 mm. Two-axis tilt (roll and pitch), COP, and platform position were collected at 100 Hz. Additional details about the perturbations can be found in [28]. The average control inputs to the platform from the experimental study were used in the simulations described below.

### Model parameterization

The model was parameterized using experimental and anthropometric data from six subjects with bilateral vestibular hypofunction who participated in a study aimed at characterizing the effect of multi-directional vibrotactile biofeedback on postural stability during discrete multidirectional support surface perturbations [28]. Detailed information about the subjects can be found in [14,28].

The average subject height and mass was 1.78 m±0.09 m (SD) and 86 kg ±5.4 kg (SD), respectively. Body link moments of inertia were calculated based on standard formulas for the cylinders and ellipsoids used to approximate the shape of the subjects’ legs, torsos, and heads. The masses and lengths of the body links were found using percent-of-total-weight [44] and percent-of-total-height [45] data.

Postural controller parameters were found by minimizing the integral of the square of the sum of the normalized differences between the experimental and simulated trajectories of the AP and ML sway and COP data. Normalization was performed using the corresponding peak experimental quantities. Hence, the optimizations sought to minimize the following function

(7) $$J=\int {\left(\frac{\left|\phi_{\exp}^{S}-\phi_{\mathrm{sim}}^{S}\right|}{\max\left|\phi_{\exp}^{S}\right|}+\frac{\left|\phi_{\exp}^{C}-\phi_{\mathrm{sim}}^{C}\right|}{\max\left|\phi_{\exp}^{C}\right|}+\frac{\left|\mathrm{CO}P_{\exp}^{S}-\mathrm{CO}P_{\mathrm{sim}}^{S}\right|}{\max\left|\mathrm{CO}P_{\exp}^{S}\right|}+\frac{\left|\mathrm{CO}P_{\exp}^{C}-\mathrm{CO}P_{\mathrm{sim}}^{C}\right|}{\max\left|\mathrm{CO}P_{\exp}^{C}\right|}\right)}^{2}\mathrm{dt}$$

where *ϕ* represents sway, *COP* is the center of pressure, subscripts exp and sim denote experimental and simulation data, respectively, and superscripts *S* and *C* denote the sagittal and coronal planes. AP sway was not included in Eq. (7) for 90° perturbations, and ML sway was not included for 180° perturbations, because the model exhibits a unidirectional response in these cases. Different control gains were used for the three perturbation directions to ensure best fit.

The bipedal stance model parameters were held constant while tuning the biofeedback model parameters for the 4-, 8-, and 16-column display models using the averaged experimental data from the 4-, 8-, and 16-column display configuration studies, respectively, and minimizing the same objective function given in Eq. (7). Only the 225° perturbation direction was considered for the 8- and 16-column displays, because they are identical to the 4-column display for the 90° and 180° perturbation directions.

A 1×2 configuration was also considered to determine if a configuration simpler than those tested experimentally could be as effective, recognizing that such a configuration could correlate with fewer electromechanical components and lower device cost. In this configuration, the two columns were assumed to be aligned with the AP axis. The parameters for this configuration were tuned using the 3×4 experimental data. The 90° perturbation direction was not considered, since the 1×2 configuration does not provide any feedback in the ML direction.

Minimizations were done by a parameter sweep in the design space followed by using the best point as an initial guess for an optimization with the Perpendicular Search approach. In cases where the design space was large, such as the 225° perturbations due to the simultaneous tuning in both sagittal and coronal controllers, a sensitivity analysis was performed to reduce the parameter sweep space for tractable computation. However, all of the parameters were included in the subsequent optimization.

### Model validation metrics

The following metrics were used to quantify the model’s quality of fit:

1. Average error: absolute value of the instantaneous difference between the experimental response and the simulated response averaged over 3 s after perturbation; values near zero indicate a good fit.

2. Cross-correlation value: cross-correlation value between the experimental response and the simulated response over 3 s after perturbation; values near 1.0 indicate a good fit.

The duration of the time window was chosen as 3 s to capture the entire transient response following perturbation. The transient part of the response is of interest, because in the steady state the model returns to the initial states of zero sway and COP.

## Results

**Figure 4:**
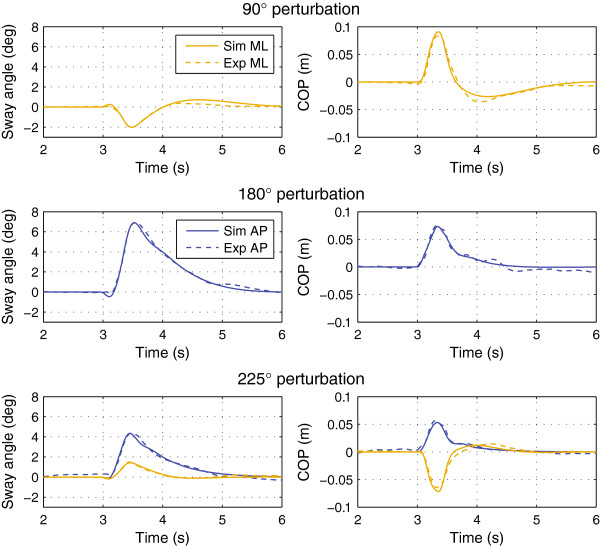
Average experimental and simulated responses without biofeedback.

**Figure 5:**
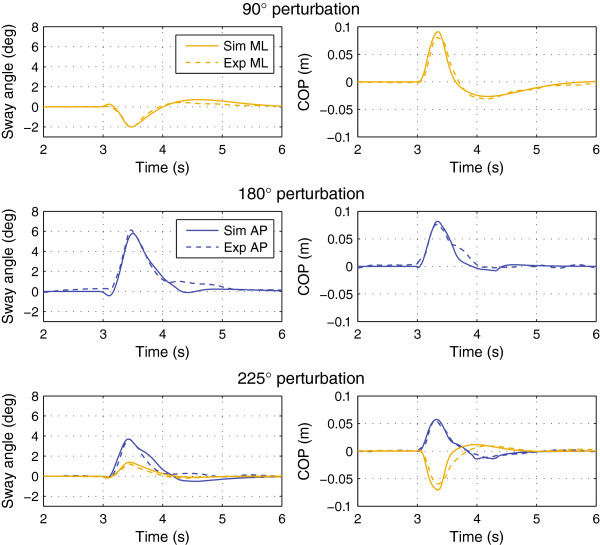
Average experimental and simulated responses for the 3×4 display configuration.

**Figure 6:**
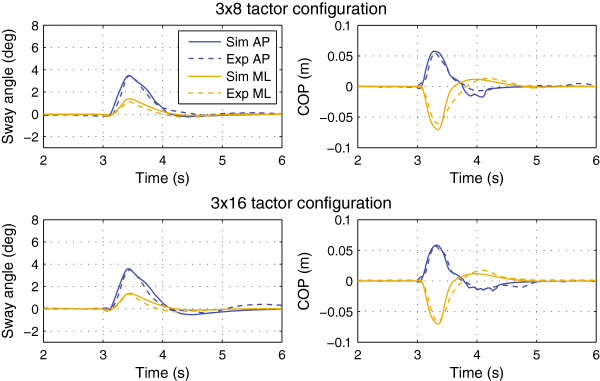
**Average experimental and simulated responses for the 3×8 and 3x16 display configurations.** Plots are for the 225° perturbation direction.

**Figure 7:**
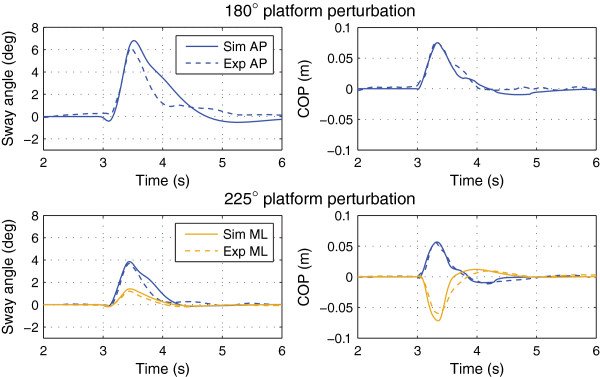
Simulated responses for the 1×2 configuration.

Figure 4 shows the experimental and simulated trajectories for the tactors-off case. Figures 5 and 6 show the trajectories for the 3×4, 3×8, and 3×16 display configurations. Figure 7 shows the simulated 1×2 display configuration and 3×4 experimental trajectories for the 180° and 225° perturbation directions. The average error and cross-correlation values are summarized in Table 1.

**Table 1:** Model validation metrics (average error and cross-correlation values) across display configurations and perturbations

	**Average error (Sway)**			
	**AP**		**ML**	
	**180°**	**225°**	**225°**	**90°**
**Tactors off**	0.16°	0.19°	0.09°	0.19°
**1×2**	0.85°	0.27°	0.11°	-
**3×4**	0.33°	0.36°	0.10°	0.18°
**3×8**	-	0.21°	0.14°	-
**3×16**	-	0.30°	0.12°	-
	**Cross-correlation value (Sway)**			
	**AP**		**ML**	
	**180°**	**225°**	**225°**	**90°**
**Tactors off**	0.998	0.99	0.97	0.95
**1×2**	0.94	0.97	0.98	-
**3×4**	0.98	0.95	0.98	0.96
**3×8**	-	0.98	0.95	-
**3×16**	-	0.97	0.93	-
	**Average error (COP)**			
	**AP**		**ML**	
	**180°**	**225°**	**225°**	**90°**
**Tactors off**	0.47 cm	0.21 cm	0.33 cm	0.46 cm
**1×2**	0.49 cm	0.26 cm	0.51 cm	-
**3×4**	0.46 cm	0.23 cm	0.51 cm	0.42 cm
**3×8**	-	0.34 cm	0.49 cm	-
**3×16**	-	0.27 cm	0.42 cm	-
	**Cross-correlation value (COP)**			
	**AP**		**ML**	
	**180°**	**225°**	**225°**	**90°**
**Tactors off**	0.98	0.99	0.98	0.98
**1×2**	0.97	0.98	0.93	-
**3×4**	0.96	0.99	0.93	0.98
**3×8**	-	0.97	0.92	-
**3×16**	-	0.98	0.95	-

Results for all display configurations, with the exception of the 1×2 case, were obtained by fitting the model to the corresponding experimental data. The model for the 1×2 display configuration was fit to the experimental data from the 3×4 case. All parameters obtained for the tactors-off condition were held fixed in the tactors-on configurations.

**Figure 8:**
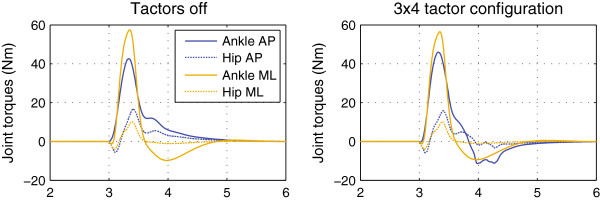
Simulated joint torque trajectories for the 225° perturbation.

While obtaining these fits, the maximum ankle and hip torques were observed to be approximately 75 N·m and 25 N·m, respectively. As an example, joint torque trajectories for the 225° perturbation direction are shown in Figure 8 for the tactors-off and 3×4 configurations. During the tactors-on conditions, the maximum additional ankle and hip torques due to biofeedback were about 8 N·m and 3 N·m, respectively. It was observed that no additional coronal torques were needed to obtain the best fits in the tactors-on conditions, including the 90° perturbations. The joint torques obtained by fits to the model were biomechanically feasible [46-48] and consistent with previously reported values [49].

Based on these results, the bipedal stance model comprising a multibody model and two independent full-state-feedback controllers for the AP and ML directions was considered valid for representing human stance during discrete multidirectional support surface perturbations, and the biofeedback model was considered valid for capturing the effect of vibrotactile biofeedback on balance.

## Discussion

Sensory reweighting [11] and sensory addition [16] have previously been described as potential mechanisms by which biofeedback devices supplement native sensory inputs to inform corrective motor torques and thereby decrease postural sway. Sensory reweighting is a general term used to describe both the real-time (i.e., while biofeedback device is being worn during a single training session) and long-term/plastic (i.e., post extensive training in the absence of wearing the biofeedback device) effects of supplemental information on the gains of native sensory inputs. In other words, the improved quality of the information about the body’s motion with respect to the gravito-inertial vector supplied by the non-native supplemental channel of information would result in an increased reliance on native inputs that correlate highly with this information. This approach infers upstream integration of the supplemental information within the central nervous system (CNS) (e.g., non-native information from the biofeedback device may be integrated with native inputs upstream within the multimodal sensory association areas in the cerebral cortex). However, to date, there is limited or no evidence in the literature to support either the real-time or long-term/plastic sensory reweighting scenarios.

Sensory addition is based on the notion that the information from the non-native channel is added to the information from native channels, with the gains of the native channels remaining unchanged. The modeling results reported by Goodworth et al. [16] suggest that the information obtained from their vibrotactile biofeedback device added to the native vestibular and proprioceptive feedback without changing the reliance on the native sensory inputs (i.e., the vestibular and proprioceptive gains remained unchanged). However, it should be noted that this approach also requires upstream integration of the supplemental information with the native inputs within the CNS.

This paper contributes to the literature by integrating biofeedback into a postural control model as an additive motor torque. Our approach integrates biofeedback on the torque side, unlike sensory reweighting or addition approaches that integrate biofeedback on the sensory side (i.e., within the Sensory Integration block in Figure 2). Our approach is important for three reasons. First, there is a lack of evidence for either real-time or long-term sensory reweighting. Second, the device used in this and our previous studies [7,11,14,16,18], which requires subjects to produce volitional (cognitively processed) responses to the supplemental information provided, suggests that it is more realistic to model the addition of the non-native body motion cues further downstream within the CNS, such as within the motor association cortex or primary motor cortex. Third, this approach is compatible with single or multiple degree-of-freedom representations of the body, multiple sensory integration models [16,29,33,34] (although not explored in this study), and various feedback modalities. Although both the sensory addition biofeedback model of Goodworth et al. and the additive torque biofeedback model described herein fit their respective experimental data well, we believe that the flexibility and scalability of the additive torque biofeedback model make it more suitable for a wide range of applications.

While looking for the simplest model to represent human stance in this study, we also considered a single-link representation. However, even though this representation was reported to be successful by Goodworth et al. [16], we found that it failed to adequately capture the experimental responses we considered. Specifically, the AP sway average errors were about nine times larger than those obtained with the two-link model. We postulate that the difference in adequacy of the single-link representation may be due to the difference in the types of perturbations. In particular, Goodworth et al. used continuous rotary platform perturbations, whereas we obtained our experimental data using discrete translational support surface perturbations. Hence, a direct comparison with the sensory addition model was not readily possible.

The two-link biomechanical model with associated controllers and biofeedback scheme is capable of capturing experimental response trajectories with low average errors and high cross-correlation values in both the AP and ML directions for all perturbation directions and device display spatial resolutions. Since the optimal fits to experimental data were obtained without the need for any coronal biofeedback torques, we conclude that the benefit of increasing the display resolution is questionable. Thus, our simulations support the experimental findings [14,28] that higher resolution displays do not necessarily correspond to better performance, possibly due to: 1) the larger effective stiffness in the ML direction than in the AP direction; and 2) the feedback display scheme. Specifically, the multidirectional perturbations elicited greater body movement in the AP than in the ML direction. This phenomenon, coupled with the “nearest neighbor” display scheme used in this paper, means that displayed information typically aligns with the AP direction for all display resolutions. Thus, the increased resolution has a negligible effect.

In fact, the simulation study with the 1×2 configuration showed that this configuration can match the experimental data for the 225° perturbation well and hence can be as effective as the 3×4 configuration for the 225° perturbation. However, for the 180° perturbation, the body sway fit obtained for the 1×2 configuration was not as good as the other fits (see Table 1), possibly because the 180° perturbation causes a larger body sway amplitudes. Therefore, a higher number of rows could be beneficial for larger sway amplitudes. These results should encourage further studies with lower display resolutions [50]. In the simulations for the 3×4, 3×8, and 3×16 configurations we observed that no biofeedback torque was generated in the hips when the first row of the device was active; instead, it caused biofeedback torques only in the ankles, whereas activation of the second row caused biofeedback torques in the hips. These observations align with previous observations that standing human subjects employ the “ankle strategy” for small perturbations and the “hip strategy” for larger perturbations [33].

In the simulations for the 3×4, 3×8, and 3×16 configurations, we observed that activation of the first row of the device caused biofeedback torques only in the ankles, whereas activation in the second row caused biofeedback torques also in the hips. These observations align with previous observations that standing human subjects employ the “ankle strategy” for small perturbations and the “hip strategy” for larger perturbations [33].

The fact that the model can account for the experimental tactors-on responses without changing the parameters of the full-state-feedback controllers of posture concurs with previous findings that biofeedback does not necessarily have to act through sensory substitution, or cause sensory reweighting to have an impact on real-time balance performance [16].

This paper’s limitations are as follows. A strict focus on perturbed stance indicates that the model and results may not be immediately transferable to studies of gait. Our model is deterministic in nature and thus does not capture the variability of the response in its current form. Because the model was developed and validated for small perturbations that do not elicit a need to move the arms or the feet and do not elicit nonlinear postural responses, studies with more severe perturbations will likely require more complicated models with nonlinear control schemes. Finally, the model does not consider the cognitive load associated with using the feedback device.

We believe that our biofeedback modeling approach has broad applicability within the field of balance rehabilitation. While this study considered a specific (vibrotactile) biofeedback device, the model is not limited to this feedback modality. Our model is easily modified to capture postural responses to auditory, visual, electrotactile, or multimodal feedback displays by incorporating different reaction time constants (*τ*_*F*_) and feedback gains (*K*_*F*_). Although we have illustrated the model’s utility for studying display resolution, we suggest that the approach can be used to study different device feedback algorithms and tactor activation schemes. The application of the model can also be extended to a wider range of subject groups, e.g., pregnant, obese, and aging populations. Such studies are beyond the scope of this paper, but are important directions for future research.

## Conclusions

The effect of biofeedback on body sway trajectories during perturbed stance can be accounted for by modeling the biofeedback as a torque signal that is added to the existing joint torques generated by the postural controller. Unlike the sensory addition approach, this torque addition approach is independent of the way the body, sensory integration, and postural control are modeled and hence provides a scalable method to integrate biofeedback. The proposed model suggests that biofeedback can work without necessarily requiring a sensory reweighting or substitution. For the specific validation study performed, the model also suggests that increased resolution in vibrotactile biofeedback displays does not necessarily lead to better performance in terms of reduced body sway. In fact, providing feedback in the sagittal plane may be adequate even during small multidirectional perturbations.

## Abbreviations

AP: Anterior-posterior; CNS: Central nervous system; COP: Center of pressure; ML: Medial-lateral.

## Competing interests

The authors declare that they have no competing interests.

## Authors’ contributions

TE conceived the study, performed the modeling, simulations, and analysis, and drafted the manuscript. KHS conceived the study, provided the experimental data, performed the analysis, and drafted the manuscript. Both authors read and approved the final manuscript.
